# A hidden twist: Twiddler’s syndrome presenting as inappropriate shock in a subcutaneous implantable cardioverter-defibrillator

**DOI:** 10.1093/ehjcr/ytag324

**Published:** 2026-05-15

**Authors:** Rafael Cantisán Campillos, Paloma Pérez Espejo, David Antonio Chipayo, Javier Fernández Portales

**Affiliations:** Cardiology Department, San Pedro de Alcántara Hospital, Avenida Pablo Naranjo Porras, s/n, Cáceres 10003, Spain; Cardiology Department, San Pedro de Alcántara Hospital, Avenida Pablo Naranjo Porras, s/n, Cáceres 10003, Spain; Cardiology Department, San Pedro de Alcántara Hospital, Avenida Pablo Naranjo Porras, s/n, Cáceres 10003, Spain; Cardiology Department, San Pedro de Alcántara Hospital, Avenida Pablo Naranjo Porras, s/n, Cáceres 10003, Spain

We present a 59-year-old male with chronic ischaemic heart disease and single-vessel disease of the left anterior descending artery. Following percutaneous revascularization, he developed severe left ventricular (LV) dysfunction, and a transvenous implantable cardioverter-defibrillator (ICD) was implanted for primary prevention. Due to a subsequent system infection, the device was extracted; as the patient had no indication for antibradycardia pacing or resynchronization therapy and to avoid a new central venous access, a subcutaneous ICD (S-ICD) was implanted (*Panel A*).

Thirty days after the S-ICD implant procedure, the patient reported a perceived shock without syncope or associated palpitations. Device interrogation showed inappropriate shock due to oversensing of noise in the ventricular canal with critically reduced shock impedance (18 Ω) (*Panel B*). Chest X-ray demonstrated complete displacement of the subcutaneous electrode, retracted from its parasternal course (*Panel C*). Given the inappropriate discharge due to mechanical failure, device explantation and reimplantation were performed (see Supplementary material online, *Video S1*). Intraoperatively, dehiscence of the generator fixation sutures to the muscle plane was observed (*Panel D*), consistent with Twiddler’s syndrome. Since the S-ICD electrode lacks active endovascular fixation, generator rotation directly tractioned the tunnelled lead, causing its complete retraction. A new S-ICD was implanted using the three-incision technique, with the lead tunnelled along the left parasternal line and securely anchored. The generator was firmly affixed to the muscular fascia with two non-absorbable sutures (*Panel E*). Fluoroscopy confirmed correct positioning (*Panel F*), appropriate sensing vectors were obtained, and ventricular fibrillation induction demonstrated effective cardioversion. The patient recovered uneventfully and remains asymptomatic.

This case illustrates the rare occurrence of Twiddler’s syndrome in an S-ICD. Few cases have been reported,^1,2^ underscoring the importance of meticulous fixation^3^ to muscle plane and preventive strategies, such as non-absorbable sutures and the use of a third suprasternal incision technique, to reduce the risk of this complication.

**Figure:**
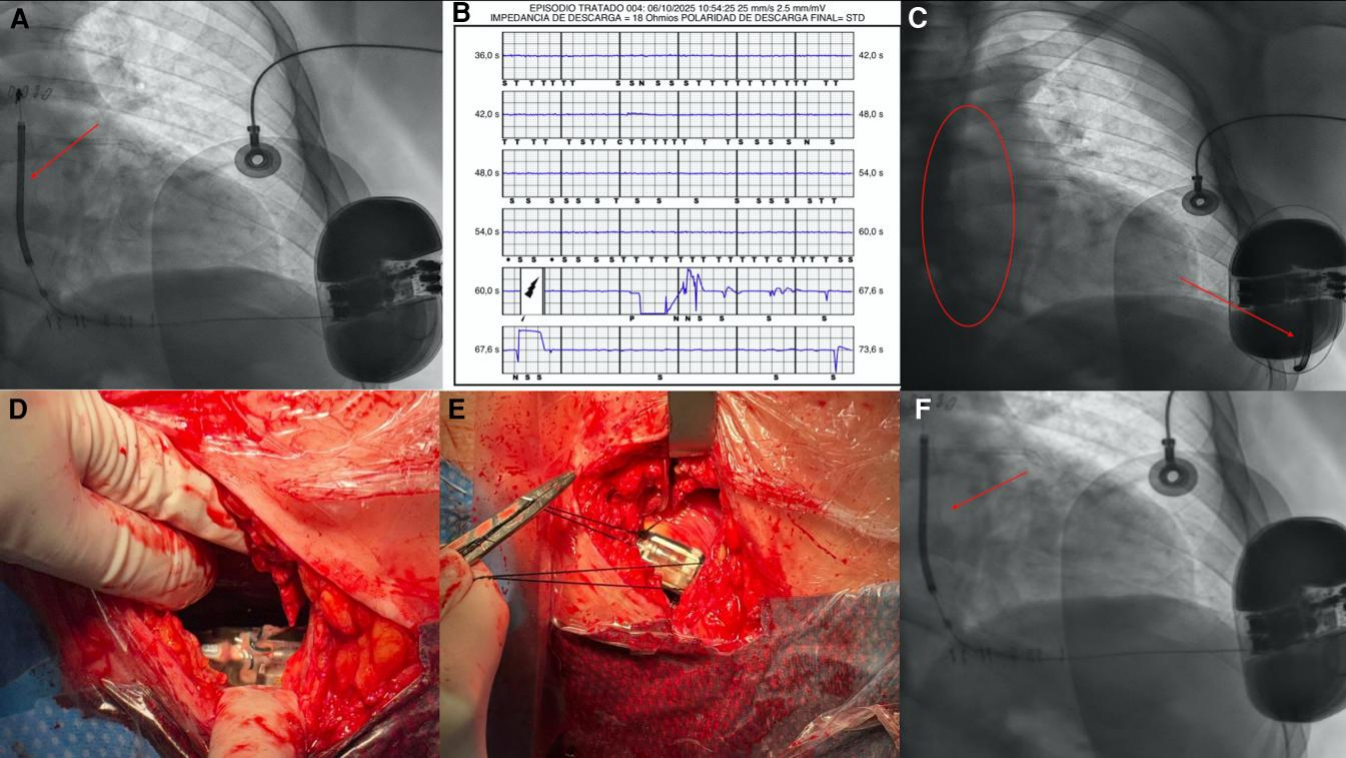


## Supplementary Material

ytag324_Supplementary_Data

## Data Availability

Data cannot be shared for ethical/privacy reasons. The data underlying this case report originate from the secure electronic medical record system of our hospital and contain sensitive patient information. Due to institutional data protection policies and to safeguard patient privacy, these data cannot be made publicly available. The data will be shared on reasonable request to the corresponding author, subject to approval by the Hospital Ethics Committee.

## Notes

**Consent:** Written informed consent was obtained from the patient for publication of this case report and the accompanying anonymized information, in accordance with COPE guidelines. The patient provided written consent for the acquisition of all imaging data and for the subsequent publication of this case, ensuring patient anonymity is protected.
